# The Adipose-Derived Stem Cell and Endothelial Cell Coculture System—Role of Growth Factors?

**DOI:** 10.3390/cells10082074

**Published:** 2021-08-13

**Authors:** Dominik Steiner, Hilkea Mutschall, Sophie Winkler, Raymund E. Horch, Andreas Arkudas

**Affiliations:** Department of Plastic and Hand Surgery, University Hospital of Erlangen, Friedrich-Alexander University Erlangen-Nürnberg (FAU), 91054 Erlangen, Germany; hilkea.mutschall@gmail.com (H.M.); sophiewinkler25@googlemail.com (S.W.); raymund.horch@uk-erlangen.de (R.E.H.); andreas.arkudas@uk-erlangen.de (A.A.)

**Keywords:** bone tissue engineering, ADSC, HUVEC, VEGFA165, BMP2

## Abstract

Adequate vascularization is a fundamental prerequisite for bone regeneration, formation and tissue engineering applications. Endothelialization of scaffold materials is a promising strategy to support neovascularization and bone tissue formation. Besides oxygen and nutrition supply, the endothelial network plays an important role concerning osteogenic differentiation of osteoprogenitor cells and consecutive bone formation. In this study we aimed to enhance the growth stimulating, proangiogenic and osteogenic features of the ADSC and HUVEC coculture system by means of VEGFA165 and BMP2 application. We were able to show that sprouting phenomena and osteogenic differentiation were enhanced in the ADSC/HUVEC coculture. Furthermore, apoptosis was unidirectionally decreased in HUVECs, but these effects were not further enhanced upon VEGFA165 or BMP2 application. In summary, the ADSC/HUVEC coculture system per se is a powerful tool for bone tissue engineering applications.

## 1. Introduction

The treatment of large volume bone defects due to trauma, oncologic surgery or infection can be challenging. The application of autologous bone still represents the current gold standard for the reconstruction of large volume bone defects [1]. Dependent on the defect size and/or entity, the use of vascularized bone grafts becomes necessary [2]. However, the transplantation of autologous bone tissue can be limited due to the defect size and the corresponding donor site morbidity. To minimize donor site morbidity, allogenic, xenogenic and alloplastic bone replacement materials such as demineralized bone matrix or bioactive glass have been introduced into clinical practice [3,4]. However, these transplants often lack the osteogenic, osteoconductive and osteoinductive potential of autologous bone grafts. Furthermore, these transplants are less suitable for larger defects due to the lack of sufficient vascularization. An elegant strategy to circumvent these limitations is the generation of bioartificial vascularized bone grafts based on the principles of tissue engineering [5]. Besides appropriate scaffold materials, the bone forming cells, as well as a sufficient vascularization strategy, seem to be the bottleneck for bone tissue engineering. In the past, different sources for the isolation, cultivation and expansion of osteoprogenitor cells have been identified. Adipose derived stem cells (ADSCs) represent a promising cell source due to their easy isolation and cultivation with only a few side effects. Furthermore, ADSCs can be differentiated into osteoprogenitor cells by the application of osteogenic supplements such as dexamethasone and 1,25-dihydroxyvitamine D3 [6]. The endothelialization of scaffolds is a strategy to facilitate vascularization of tissue engineered constructs and to enhance cell survival as well as tissue formation [7,8]. Besides the preservation of tissue homeostasis, the microvascular network plays an important role in signal transduction. We and others have shown that endothelial cells can stimulate proliferation and cell survival of cocultured osteoprogenitor cells [9,10,11]. Moreover, endothelial cells stimulate the osteogenic differentiation of osteoprogenitor cells as indicated by increased matrix mineralization and the production of osteogenic markers. Alkaline phosphatase (ALP) is a prominent early osteogenic marker, which is upregulated in osteoprogenitor cells upon 48–72 h of cocultivation with endothelial cells [12,13]. In a previous study we have been able to prove that coculturing ADSCs and human umbilical vein endothelial cells (HUVECs) in a ratio of 1:1 displays the best effects with regard to proliferation, cell survival and osteogenic differentiation. This study intended to enhance the positive effects of the ADSC/HUVEC coculture system by the application of BMP2 and VEGFA165.

## 2. Materials and Methods

### 2.1. Cell Culture

HUVECs were purchased from PromoCell (Heidelberg, Germany) and cultured in Endothelial Cell Growth Medium (ECGM) (PromoCell, Heidelberg, Germany). ECGM contained Endothelial Cell Basal Medium (ECBM) and supplements (PromoCell), 10% FCS superior, 100 U/mL Penicillin and 100 µg/mL Streptomycin (Biochrom, Berlin, Germany). According to an established protocol, ADSCs were isolated from five patients undergoing autologous breast reconstruction with an abdominal free flap [14]. The ethics committee approved the isolation of ADSCs [AZ: 126_16] and the patients gave informed consent. ADSCs were cultured and expanded in MEMa (Gibco, Paisley, UK) supplemented with 10% FCS superior, 100 U/mL Penicillin and 100 µg/mL Streptomycin (all supplements from Biochrom). Osteogenic differentiation of ADSCs was induced prior to the experiments. For this purpose, ADSCs were cultured in ECGM medium containing an additional 1 × 10^−8^ M dexamethasone, 50 µg/mL L-ascorbic acid, 10 mM glycerophosphate and 0.01 µM 1,25-dihydroxyvitamine D3 (all supplements purchased from Sigma, Steinheim, Germany) for 14 days [6,13]. For the following experiments, three experimental groups were performed. All groups comprised the untreated ADSC and HUVEC monoculture as well as the ADSC/HUVEC coculture in a ratio of 1:1. The cells in the control group were cultured in osteogenic modified ECGM. The other two experimental groups were either cultured in osteogenic modified ECGM supplemented with VEGFA165 (100 ng/mL) or BMP2 (60 µg/mL). VEGFA165 was purchased from R&D Systems (Minneapolis, MN, USA) and BMP2 in the form of InductOs was purchased from Medtronic (Heerlen, Netherlands). The growth factors were reconstituted as recommended by the manufacturers. Cells were seeded in a density of 5000 cells/cm^2^ in standard two-dimensional cell culture plates in an humidified atmosphere at 37 °C and 5% CO_2_. The media were changed twice a week.

### 2.2. Immunoselection Using Magnetic Beads

Depending on the experimental setup, cells were detached from the cell culture dishes and negative immunoselection was performed after 3 or 7 days. Briefly, the detached cells were resuspended in 1 ml PBS containing 0.1% BSA. Magnetic beads (25 µL) coated with a CD31 antibody (Invitrogen, Waltham, MA, USA) were added and incubation was performed for 20 min at 4 °C Afterwards, cell separation was performed with a magnetic separator (DynaMgTM, Invitrogen, Oslo, Norway) and each cell type was used for the further experiments.

### 2.3. Cell Proliferation Assay

After 7 days, cells were detached from the cell culture plate, separated using negative immunoselection and counted. The cell number was counted with a TC20TM automated cell counter (BIO-RAD, Hercules, CA, USA).

### 2.4. Cell Death Detection ELISA

After 7 days of cell culture, apoptosis was measured and quantified using the well-established cell death detection ELISA (Sigma, Steinheim, Germany). Briefly, cells were lyzed in 200 µL lysis buffer and centrifuged at 200× *g* for 10 min. Thereafter, 20 µL of the supernatant were pipetted on a microplate coated with an anti-histone antibody. After a washing step, a peroxidase-labelled anti-DNA antibody was added. The enzyme reaction was induced with ABTS substrate (2,2′-Azino-di [3-ethylbenzthiazolin-sulfonat]) and absorbance was measured at 405 nm.

### 2.5. Matrigel Assay

A matrigel assay was performed to assess the influence of VEGFA165 and BMP2 on the angiogenic characteristics of the ADSC/HUVEC coculture system. Briefly, the wells of a µ-Slice (Ibidi, Martinsried, Germany) were filled with 10 µL matrigel (Corning, Kaiserslautern, Germany). After the matrigel polymerized, 10,000 cells per well were added and incubation occurred for 4 h. Vital cells were marked with calcein (Sigma). The newly formed vessel network was analyzed with Angiogenesis Analyzer (ImageJ Version 2 NIH, Bethesda, MD, USA).

### 2.6. RNA Isolation, Reverse Transcription and Quantitative Real Time PCR

The early osteogenic differentiation marker alkaline phosphatase (ALP) was assessed using quantitative real time polymerase chain reaction (qRT-PCR). After 3 days of cell culture, the cells were detached and separated, and total RNA was extracted with the RNeasy^®^ Mini Kit (QIAGEN, Hilden, Germany) following the manufacturer’s instructions. cDNA synthesis was performed with 1 µg RNA using the QuantiTect^®^ Reverse Transcription Kit (QIAGEN) according to company guidelines. For qRT-PCR, 25 ng cDNA, SsoAvancedTM Universal SYBR^®^ Green Supermix (BIO-RAD) and the primers for ALP and RPL13a (reference gene) were used. Each sample was normalized to RPL13a and the relative standard curve method was used for analysis [15].

### 2.7. Statistical Analysis

Statistical analysis was performed with Graph Pad Prism 7.00 (Graph Pad Software, San Diego, CA, USA). After testing for normal distribution, analysis of variance (ANOVA) was performed with Tukey test for multiple comparison. *p* values ≤ 0.05 were defined as statistically significant.

## 3. Results

### 3.1. Proliferation

**Figure 1:**
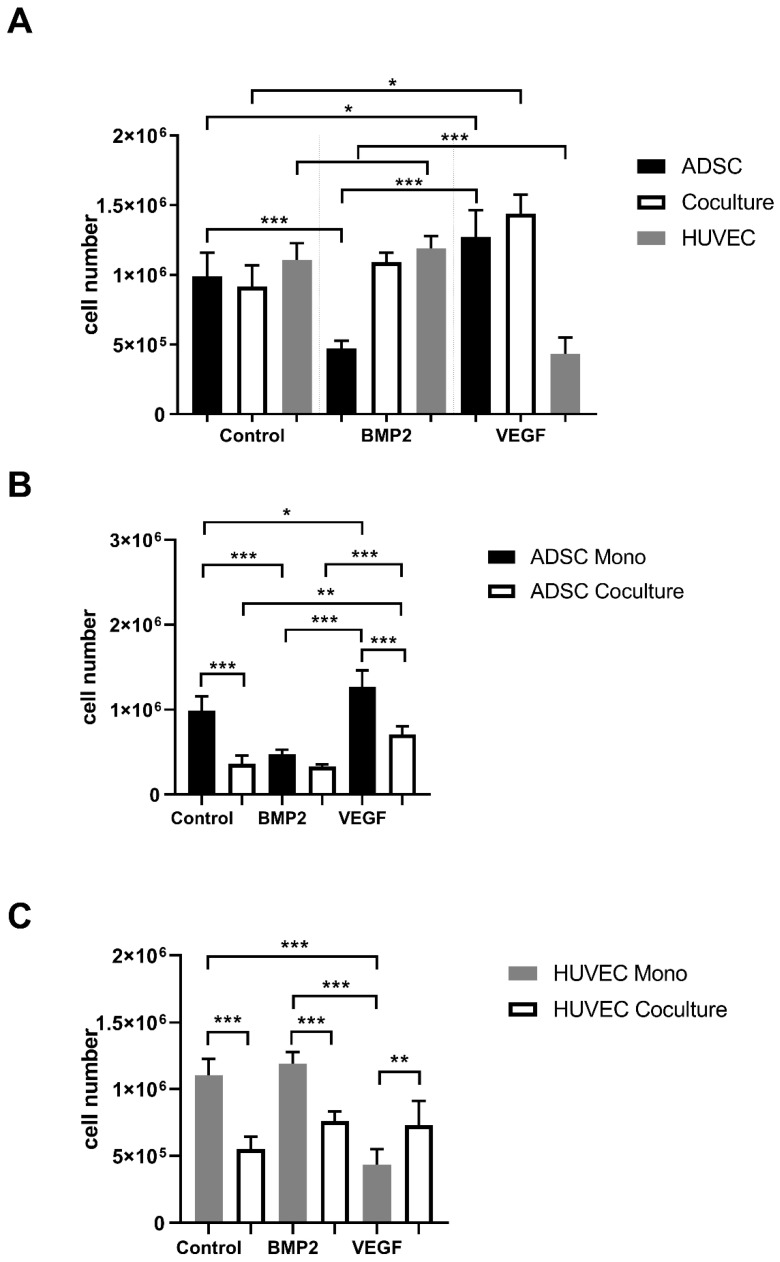
Proliferation rate of ADSCs and HUVECs under mono- and coculture conditions. After adding VEGFA165, a statistically significant increase of the total cell number was detected in the ADSC monoculture as well in the coculture group (**A**). Using negative immunoselection, the effects of BMP2 or VEGFA165 were observed cell type specific (**B**,**C**). Statistically significant differences between the experimental groups are indicated for * *p* ≤ 0.05, ** *p* ≤ 0.01 and *** *p* ≤ 0.001. Proliferation rate of ADSCs and HUVECs under mono- and coculture conditions. After adding VEGFA165, a statistically significant increase of the total cell number was detected in the ADSC monoculture as well in the coculture group (**A**). Using negative immunoselection, the effects of BMP2 or VEGFA165 were observed cell type specific (**B**,**C**). Statistically significant differences between the experimental groups are indicated for * *p* ≤ 0.05, ** *p* ≤ 0.01 and *** *p* ≤ 0.001.

To assess the impact of VEGFA165 and BMP2 on proliferation, a proliferation assay was performed. After 7 days, cells were detached, separated and counted individually. Considering the total cell number, no significant increase was measured in the coculture compared to the ADSC monoculture in the control group. However, a statistically significant increase was observed in the ADSC monoculture and coculture after adding VEGFA165 compared to the control group (*p* ≤ 0.05). BMP2 did not affect the cell number in the coculture and HUVEC monoculture compared to the control group. A statistically significant lower cell number can be found in the ADSC monoculture treated with BMP2 (*p* ≤ 0.001) compared to the control group and the ADSC monoculture treated with VEGFA165 (Figure 1A). Using negative immunoselection, the impact of VEGFA165 and BMP2 was counted cell type specific in the cocultures (Figure 1B,C). With regard to the ADSC control groups, only VEGFA165 induced a statistically significant increase of the cell number under coculture conditions (*p* ≤ 0.01). Compared to the control groups, a statistically significant lower cell number was only found in the HUVEC monoculture upon VEGFA165 treatment.

### 3.2. Apoptosis

**Figure 2:**
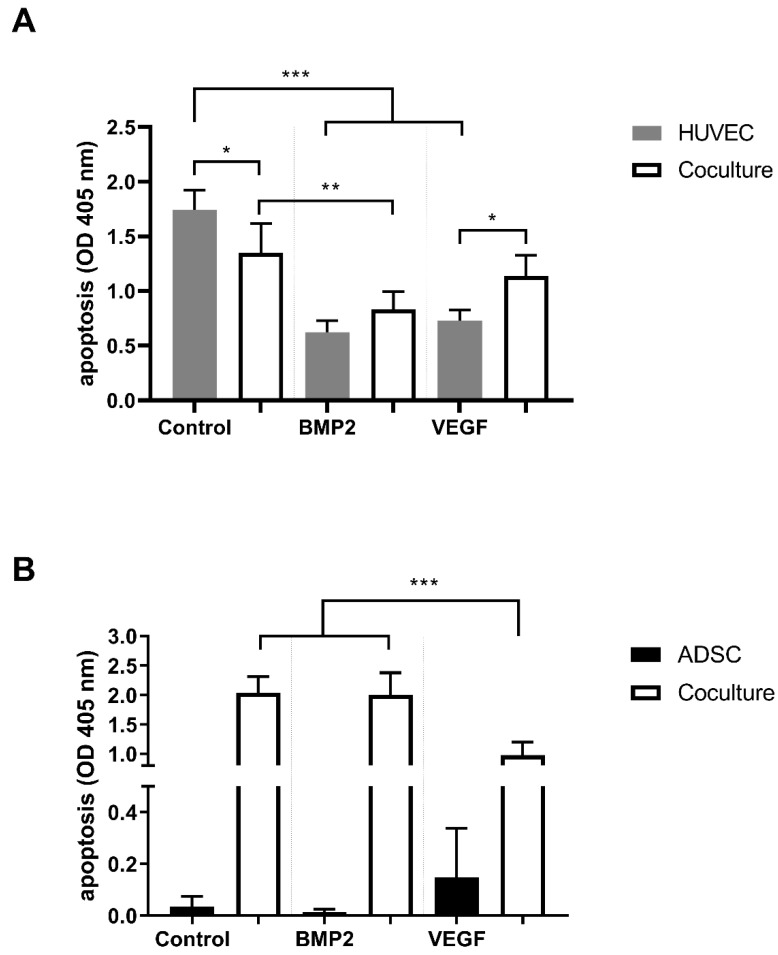
Apoptosis of ADSCs and HUVECs under mono- and coculture conditions. Coculturing reduced apoptosis in the HUVEC control group. Moreover, BMP2 and VEGFA165 reduced apoptosis in the HUVEC monocultures (**A**). VEGFA165 reduced apoptosis in the cocultured ADSCs (**B**). Statistically significant differences between the experimental groups are indicated for * *p* ≤ 0.05, ** *p* ≤ 0.01 and *** *p* ≤ 0.001.

We measured a statistically significant reduction of apoptosis in the HUVEC coculture control group (*p* ≤ 0.05). Moreover, the addition of VEGFA165 and BMP2 reduced apoptosis in monocultured HUVECs to a high extent (*p* ≤ 0.001). Conversely, only the addition of BMP2 induced a statistically significant reduction of apoptosis in cocultured HUVECs compared to the coculture control group (*p* ≤ 0.01; Figure 2A). In ADSCs, VEGFA165 and BMP2 treatment did not influence cell survival under monoculture conditions. On the other hand, VEGFA165 treatment reduced apoptosis significantly (*p* ≤ 0.001) in the cocultured ADSCs compared to the corresponding control and BMP2 group (Figure 2B).

### 3.3. Matrigel Assay

**Figure 3:**
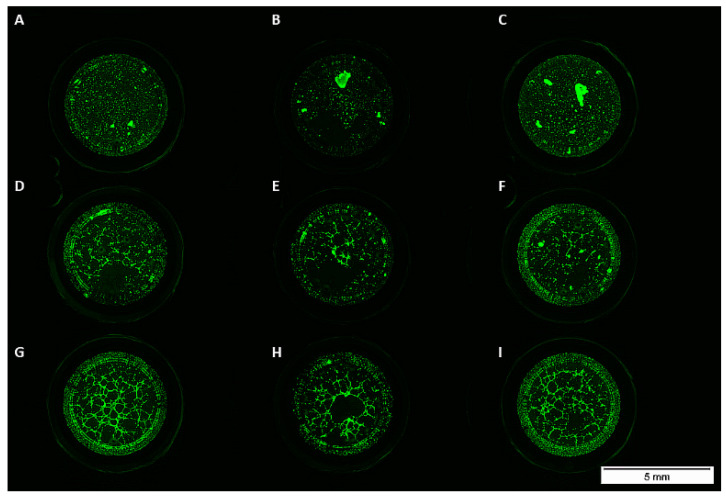
Monocultured ADSCs treated with or without BMP2 or VEGFA165 formed no tubes in matrigel (**A**–**C**). Sprouting was observed in the coculture (**D**–**F**) and HUVEC monoculture groups (**G**–**I**).

To investigate the impact of VEGFA165 and BMP2 on network formation, we performed a matrigel assay. Unlike osteogenic differentiated ADSCs, HUVECs and cocultures from all experimental groups formed tubes (Figure 3).

**Figure 4:**
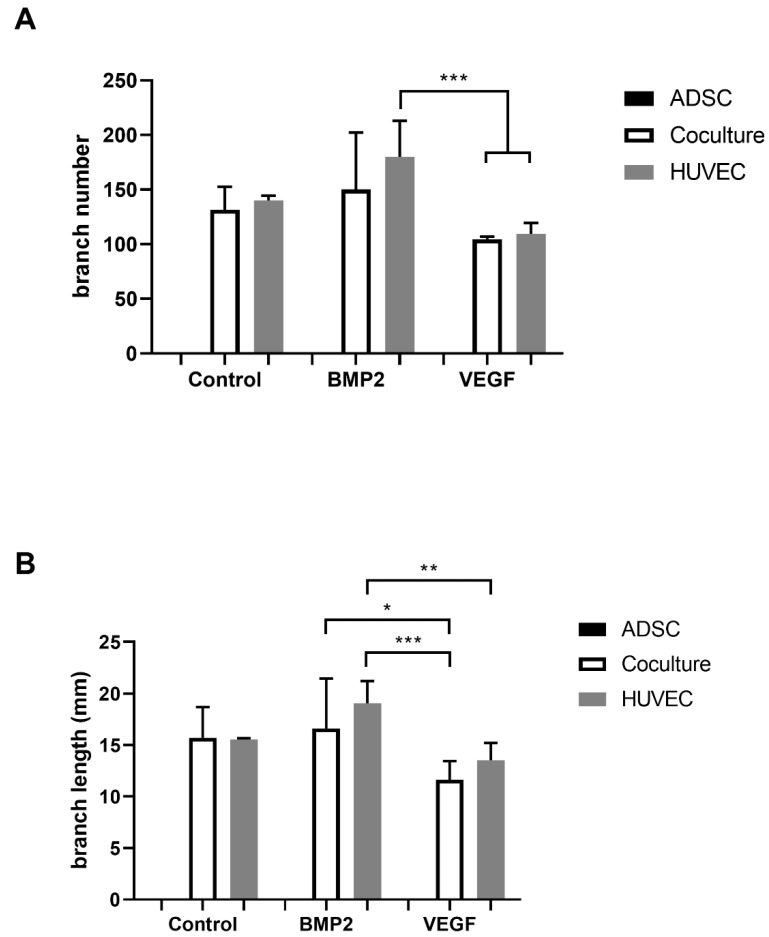
Compared to the control group, no effect of BMP2 or VEGFA165 was observed considering the branch number and length (**A**,**B**). BMP2 seems to be advantageous compared to VEGFA165 concerning the branch number and length. Statistically significant differences between the experimental groups are indicated for * *p* ≤ 0.05, ** *p* ≤ 0.01 and *** *p* ≤ 0.001.

Even the addition of VEGFA165 or BMP2 did not influence network formation of ADSCs in the monoculture (Figure 3A–C and Figure 4A,B). Compared to the control group, the addition of growth factors did not influence network formation (Figure 4A,B).

### 3.4. ALP Gene Expression

**Figure 5:**
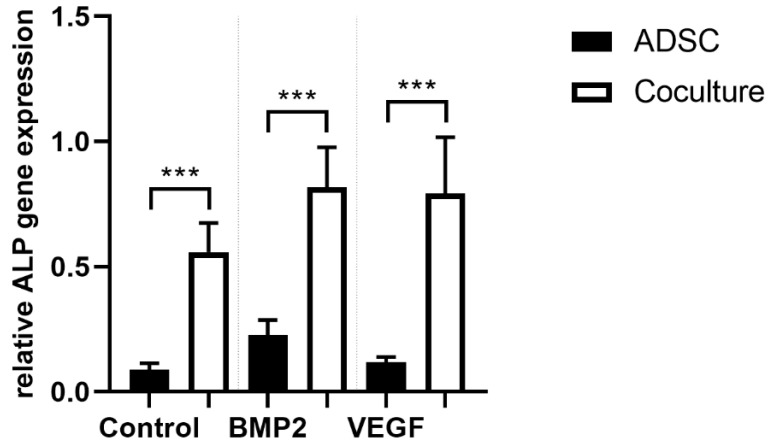
After negative immunoselection, PCR analysis was carried out in ADSCs. Alkaline phosphatase (ALP) gene expression as a surrogate parameter for osteogenic differentiation was upregulated under coculture conditions. BMP2 or VEGFA165 had no effect on ALP gene expression. Statistically significant differences between the experimental groups are indicated for *** *p* ≤ 0.001.

Alkaline phosphatase is a well-established parameter for early osteogenic differentiation. Negative immunoselection allows the specific quantification of ALP gene expression of ADSCs under coculture conditions. In all cocultures we were able to prove a statistically significant increase of ALP gene expression (*p* ≤ 0.001) compared to the corresponding monocultures. VEGFA165 and BMP2 did not change ALP gene expression in the mono- and cocultures (Figure 5).

## 4. Discussion

Bone formation and angiogenesis are two closely related processes. Inhibition of angiogenesis can lead to impaired bone formation and/or insufficient fracture healing [16,17]. Besides a sufficient microvascular network providing nutrients and oxygen, endothelial cells are critically involved in intercellular signal transduction with osteoprogenitor cells. In this regard, we [11,18] and other groups [9,12] have been able to prove that coculturing endothelial cells with osteoprogenitor cells increases cell survival, proliferation and osteogenic differentiation. In this regard, heterotypic cell contacts between endothelial cells and osteoprogenitor cells seem to be the driving force [19]. In a recent work we demonstrated that coculturing adipose derived stem cells (ADSCs) and human umbilical vein endothelial cells (HUVECs) stimulates proliferation, proangiogenic effects and osteogenic differentiation [13]. We translated our in vitro results into the rat arteriovenous loop (AV loop) model. Forming an arteriovenous fistula between the femoral vessels by means of a venous interponate, vascularization of an hydroxyapatite-fibrin matrix was surgically induced. After six weeks, we showed that the coimplantation of ADSCs and HUVECs stimulated bone formation of the hydroxyapatite-fibrin matrix [20]. Because only approximately 20% of the newly formed tissue was mineralized, we intended to enhance the osteogenic potential of the ADSC/HUVEC coculture system with the application of growth factors. Vascular endothelial growth factor A isoform 165 (VEGFA165) and bone morphogenetic protein 2 (BMP2) are well known growth factors associated with bone formation and angiogenesis [17,21,22,23]. Based on previous work and in order to translate our findings into the AV loop model, we used a concentration of 60 µg/mL BMP2 and 100 ng/mL VEGFA165 for our study [21,24,25]. After 7 days of incubation, we did not detect a growth stimulating effect in the ADSC/HUVEC coculture without growth factors. However, we were able to prove that VEGFA165 induced proliferation in ADSCs under mono- and coculture conditions compared to the control group. Interestingly, the application of VEGFA165 did not stimulate the proliferation of HUVECs under coculture conditions. Moreover, the application of VEGFA165 reduced the cell number of HUVECs under monoculture conditions compared to the control group. Based on the findings from a previous study, it is alluring to speculate that VEGFA165 concentrations higher than 200 pg/mL might have adverse effects on HUVEC proliferation [13]. From the pertinent literature, it is well-known that VEGF can stimulate the proliferation rate of ADSCs in a dose dependent manner [26]. BMP2 did not affect the proliferation rate of HUVECs neither under mono- nor under coculture conditions. Besides that, BMP2 reduced the cell number of ADSCs in the monoculture, whereas the cell number in the coculture remained unaffected. In accordance to a previous study, we were able to show that cocultivation reduced the apoptosis rate in HUVECs in the control group [13]. This effect was only unidirectional and not observed in cocultured ADSCs. The addition of BMP2 or VEGFA165 further reduced the apoptosis rate in HUVECs under monoculture conditions compared to the corresponding control group. Interestingly, this effect was not observed in cocultured HUVECs. It might be reasonable to assume that apoptosis is regulated independently of BMP2 and/or VEGFA165 in HUVECs under coculture conditions. The results from the proliferation and apoptosis experiments are in accordance with previous work demonstrating that heterotypic cell contacts between HUVECs and osteoprogenitor cells have a strong influence on proliferation and/or cell survival [11]. Given the fact that vascularization of bone tissue engineering constructs is the major limiting step for the clinical application, we tried to enhance the angiogenic potential of the ADSC/HUVEC coculture system. In accordance to a previous study, we were able to show that osteogenic differentiated ADSCs under monoculture conditions did not sprout in the matrigel assay [13]. In the HUVEC monoculture and in the coculture of ADSCs and HUVECs, we observed sprouting phenomena. Surprisingly, the addition of VEGFA165 or BMP2 had no effect on the sprouting performance compared to the control group. The pertinent literature proves that cocultivation of endothelial cells with osteoprogenitor cells stimulates the osteogenic differentiation [12,13,18,27,28]. Most often, alkaline phosphatase (ALP) is used as a marker molecule for early osteogenic differentiation. Using negative immunoselection, we demonstrated increased ALP gene expression in ADSCs after 72 h of cocultivation with HUVECs. Interestingly, the application of VEGFA165 or BMP2 did not further increase ALP gene expression in ADSCs neither in the monoculture nor in the coculture. Consistent with the pertinent literature, the role of paracrine acting molecules such as VEGFA165 or BMP2 is controversial. Heterotypic cell contacts between endothelial cells and osteoprogenitor cells or the extracellular matrix seem to play a superior role [18,29].

## 5. Conclusions

In our study, we were able to demonstrate that coculturing ADSCs and HUVECs stimulates osteogenic differentiation and proangiogenic effects. Moreover, apoptosis was decreased in HUVECs in a unidirectional manner. The addition of VEGFA165 or BMP2 did not further stimulate osteogenic differentiation, sprouting phenomena or cell survival of the ADSC/HUVEC coculture.

## Data Availability

The data presented in this study are available on request from the corresponding author.

## References

[1] Gazdag A.R., Lane J.M., Glaser D., Forster R.A. (1995). Alternatives to Autogenous Bone Graft: Efficacy and Indications. J. Am. Acad. Orthop. Surg..

[2] Willemot L., Stewart D., Lawson R. Reconstruction of an infected midshaft radius and ulna nonunion using a free vascularized fibula and medial femoral condyle flap. Microsurgery.

[3] Baldwin P., Li D.J., Auston D.A., Mir H.S., Yoon R.S., Koval K.J. (2019). Autograft, Allograft, and Bone Graft Substitutes: Clinical Evidence and Indications for Use in the Setting of Orthopaedic Trauma Surgery. J. Orthop. Trauma.

[4] Dorea H.C., McLaughlin R.M., Cantwell H.D., Read R., Armbrust L., Pool R., Roush J.K., Boyle C. (2005). Evaluation of healing in feline femoral defects filled with cancellous autograft, cancellous allograft or Bioglass. Vet. Comp. Orthop. Traumatol..

[5] Xie C., Ye J., Liang R., Yao X., Wu X., Koh Y., Wei W., Zhang X., Ouyang H. (2021). Advanced Strategies of Biomimetic Tissue-Engineered Grafts for Bone Regeneration. Adv. Healthc. Mater..

[6] Li S., Hu C., Li J., Liu L., Jing W., Tang W., Tian W., Long J. (2016). Effect of miR-26a-5p on the Wnt/Ca(2+) Pathway and Osteogenic Differentiation of Mouse Adipose-Derived Mesenchymal Stem Cells. Calcif. Tissue Int..

[7] Koob S., Torio-Padron N., Stark G.B., Hannig C., Stankovic Z., Finkenzeller G. (2011). Bone formation and neovascularization mediated by mesenchymal stem cells and endothelial cells in critical-sized calvarial defects. Tissue Eng. Part A.

[8] Zhang X., Yang J., Li Y., Liu S., Long K., Zhao Q., Zhang Y., Deng Z., Jin Y. (2011). Functional neovascularization in tissue engineering with porcine acellular dermal matrix and human umbilical vein endothelial cells. Tissue Eng. Part C Methods.

[9] Bidarra S.J., Barrias C.C., Barbosa M.A., Soares R., Amedee J., Granja P.L. (2011). Phenotypic and proliferative modulation of human mesenchymal stem cells via crosstalk with endothelial cells. Stem Cell Res..

[10] Jones A.R., Clark C.C., Brighton C.T. (1995). Microvessel endothelial cells and pericytes increase proliferation and repress osteoblast phenotypic markers in rat calvarial bone cell cultures. J. Orthop. Res..

[11] Steiner D., Lampert F., Stark G.B., Finkenzeller G. (2012). Effects of endothelial cells on proliferation and survival of human mesenchymal stem cells and primary osteoblasts. J. Orthop. Res..

[12] Hager S., Lampert F.M., Orimo H., Stark G.B., Finkenzeller G. (2009). Up-regulation of alkaline phosphatase expression in human primary osteoblasts by cocultivation with primary endothelial cells is mediated by p38 mitogen-activated protein kinase-dependent mRNA stabilization. Tissue Eng. Part A.

[13] Mutschall H., Winkler S., Weisbach V., Arkudas A., Horch R.E., Steiner D. (2020). Bone tissue engineering using adipose-derived stem cells and endothelial cells: Effects of the cell ratio. J. Cell Mol. Med..

[14] Weigand A., Boos A.M., Tasbihi K., Beier J.P., Dalton P.D., Schrauder M., Horch R.E., Beckmann M.W., Strissel P.L., Strick R. (2016). Selective isolation and characterization of primary cells from normal breast and tumors reveal plasticity of adipose derived stem cells. Breast Cancer Res..

[15] Medhurst A.D., Harrison D.C., Read S.J., Campbell C.A., Robbins M.J., Pangalos M.N. (2000). The use of TaqMan RT-PCR assays for semiquantitative analysis of gene expression in CNS tissues and disease models. J. Neurosci. Meth..

[16] Gerber H.P., Vu T.H., Ryan A.M., Kowalski J., Werb Z., Ferrara N. (1999). VEGF couples hypertrophic cartilage remodeling, ossification and angiogenesis during endochondral bone formation. Nat. Med..

[17] Street J., Bao M., deGuzman L., Bunting S., Peale F.V., Ferrara N., Steinmetz H., Hoeffel J., Cleland J.L., Daugherty A. (2002). Vascular endothelial growth factor stimulates bone repair by promoting angiogenesis and bone turnover. Proc. Natl. Acad. Sci. USA.

[18] Lampert F.M., Simunovic F., Finkenzeller G., Pfeifer D., Stark G.B., Winninger O., Steiner D. (2016). Transcriptomic Changes in Osteoblasts Following Endothelial Cell-Cocultivation Suggest a Role of Extracellular Matrix in Cellular Interaction. J. Cell Biochem..

[19] Simunovic F., Steiner D., Pfeifer D., Stark G.B., Finkenzeller G., Lampert F. (2013). Increased extracellular matrix and proangiogenic factor transcription in endothelial cells after cocultivation with primary human osteoblasts. J. Cell Biochem..

[20] Winkler S., Mutschall H., Biggemann J., Fey T., Greil P., Korner C., Weisbach V., Meyer-Lindenberg A., Arkudas A., Horch R.E. (2021). Human Umbilical Vein Endothelial Cell Support Bone Formation of Adipose-Derived Stem Cell-Loaded and 3D-Printed Osteogenic Matrices in the Arteriovenous Loop Model. Tissue Eng. Part A.

[21] Buehrer G., Balzer A., Arnold I., Beier J.P., Koerner C., Bleiziffer O., Brandl A., Weis C., Horch R.E., Kneser U. (2015). Combination of BMP2 and MSCs significantly increases bone formation in the rat arterio-venous loop model. Tissue Eng. Part A.

[22] Meng C., Su W., Liu M., Yao S., Ding Q., Yu K., Xiong Z., Chen K., Guo X., Bo L. (2021). Controlled delivery of bone morphogenic protein-2-related peptide from mineralised extracellular matrix-based scaffold induces bone regeneration. Mater. Sci. Eng. C Mater. Biol. Appl..

[23] van Houdt C.I.A., Koolen M.K.E., Lopez-Perez P.M., Ulrich D.J.O., Jansen J.A., Leeuwenburgh S.C.G., Weinans H.H., van den Beucken J. (2021). Regenerating Critical Size Rat Segmental Bone Defects with a Self-Healing Hybrid Nanocomposite Hydrogel: Effect of Bone Condition and BMP-2 Incorporation. Macromol. Biosci..

[24] Arkudas A., Pryymachuk G., Hoereth T., Beier J.P., Polykandriotis E., Bleiziffer O., Horch R.E., Kneser U. (2009). Dose-finding study of fibrin gel-immobilized vascular endothelial growth factor 165 and basic fibroblast growth factor in the arteriovenous loop rat model. Tissue Eng. Part A.

[25] Zara J.N., Siu R.K., Zhang X., Shen J., Ngo R., Lee M., Li W., Chiang M., Chung J., Kwak J. (2011). High doses of bone morphogenetic protein 2 induce structurally abnormal bone and inflammation in vivo. Tissue Eng. Part A.

[26] Chen G., Shi X., Sun C., Li M., Zhou Q., Zhang C., Huang J., Qiu Y., Wen X., Zhang Y. (2013). VEGF-mediated proliferation of human adipose tissue-derived stem cells. PLoS ONE.

[27] Villars F., Bordenave L., Bareille R., Amedee J. (2000). Effect of human endothelial cells on human bone marrow stromal cell phenotype: Role of VEGF?. J. Cell Biochem..

[28] Villars F., Guillotin B., Amedee T., Dutoya S., Bordenave L., Bareille R., Amedee J. (2002). Effect of HUVEC on human osteoprogenitor cell differentiation needs heterotypic gap junction communication. Am. J. Physiol. Cell Physiol..

[29] Steiner D., Winkler S., Heltmann-Meyer S., Trossmann V., Fey T., Scheibel T., Horch R.E., Arkudas A. (2021). Enhanced vascularization andde novotissue formation in hydrogels made of engineered RGD-tagged spider silk proteins in the arteriovenous loop model. Biofabrication.

